# A commentary on ‘Radiographic views for hand fractures – call for three-view national UK guidelines – a quality improvement study’

**DOI:** 10.1097/JS9.0000000000000678

**Published:** 2023-10-11

**Authors:** Si-Un Frank Chiu, Shih-Chieh Yang, Chong-Chi Chiu

**Affiliations:** a Department of Computer Science; b Department of Economics, Brown University, Providence, Rhode Island, USA; c Department of Orthopedic Surgery, E-Da Hospital; d School of Medicine, College of Medicine; e Department of General Surgery; f Department of Medical Education and Research, E-Da Cancer Hospital, I-Shou University, Kaohsiung, Taiwan

*Dear Editor*,

Acute phalangeal and metacarpal hand fractures are common presentations in the emergency department (ED), necessitating swift and accurate diagnosis for appropriate management. Conventional radiography remains the primary imaging modality for identifying fractures due to its accessibility and familiarity among physicians. However, the limitations of two-view radiographs, especially in complex joints like the hand and wrist, may result in missed fractures and delayed diagnoses. In light of this, the call for quality improvement in the form of national UK guidelines, as proposed by Southall *et al*.^1^, raises essential considerations among ED professionals.

Efforts have been made to develop clinical decision tools that aid in identifying patients requiring radiographic evaluation. For instance, the ‘Amsterdam Wrist Rules’ by Walenkamp *et al*. demonstrated high sensitivity (98%) and negative predictive value (90%) for wrist fractures, providing a useful screening tool. However, concerns arise regarding its low specificity and minimal reduction in unnecessary X-ray indications, potentially leading to sequelae from missed fractures. Similarly, Karaca *et al*. proposed another decision rule but faced criticism for unclear statistical methods and limited statistical significance. The absence of a universally accepted decision rule makes the recommendation for radiography even in low-risk patients by Van den Brand *et al*.^2^, as blunt finger or wrist traumas carry a considerable fracture risk.

Many studies have primarily focused on the sensitivity and specificity of imaging findings in potential fracture detection. Although conventional radiographs usually provide sufficient information to guide the treating physicians, two views are inadequate for detecting some radiographically occult fractures, like distal radius and scaphoid fractures. Thus, for joints in the extremities, especially the wrist, hand, and fingers, a three-view radiographic examination (anteroposterior, oblique, and lateral views) is often essential for accurate fracture detection. Studies indicate that a semi-supinated oblique projection can further enhance diagnostic yield for distal radius fractures^3^. Delayed diagnoses, particularly in the radiographically occult distal radius and scaphoid fractures, underscore the importance of comprehensive imaging approaches.

To avoid nonunion complications from missed fractures, physicians usually prefer initial immobilization of ambiguous injuries and typically appoint follow-up for re-evaluation. Researchers have explored the diagnostic accuracy and cost-effectiveness of advanced imaging techniques like magnetic resonance imaging (MRI), comp uted tomography (CT), and bone scans for suspected scaphoid fractures. Yin’s study showed that, in most cases in China, immediate CT or MRI is more cost-effective than follow-up radiograph-based strategies or bone scans on the 3rd day. However, a study in the United Kingdom by Jenkins *et al*. suggested that early MRI was more expensive than follow-up radiographs or early CT^4^. However, cost-effectiveness comparisons between CT or MRI and follow-up radiographs or bone scans must also account for factors like lost productivity and medicolegal disputes arising from missed fractures. Thus, when initial two-view or even three-view radiographs are normal, but there is high clinical suspicion of fracture, we recommend early imaging with CT or MRI is most appropriate.

Overburdened physicians, fatigue, and limited experience may impact diagnostic performance. Instant radiograph interpretation by specialized radiologists or automated analysis using artificial intelligence (AI) algorithms can help minimize image interpretation errors in the ED. Remarkable strides in deep learning, a subfield of AI, have demonstrated the feasibility of automating wrist fracture detection using convolutional neural networks. Some studies highlighted the superior performance of AI in wrist fracture detection compared to nonspecialized radiologists^5^. Integrating AI assistance with expert analysis is believed to enhance fracture detection sensitivity and specificity, optimizing decision-making without prolonging reading time.

Clinical hand and wrist fracture diagnosis demand meticulous attention and a comprehensive approach to avoid mismanagement of occult injuries and their severe consequences. Incorporating detailed patient history, thorough physical examination, and appropriate imaging can reduce the risk of missed fractures. Additionally, AI assistance can provide invaluable support to radiologists during trauma emergencies, mitigating radiologist burnout, reducing healthcare and lost productivity costs, ensuring patient safety, and ultimately lowering the possibility of medicolegal disputes. By embracing these strategies and advancements, healthcare professionals can elevate the standard of care for patients with acute hand and wrist trauma, ensuring timely and accurate diagnoses, fostering improved patient outcomes and quality of care. Although integrating AI assistance and radiologic expert analysis could yield the best performances at this moment, it is necessary to determine whether this combination strategy could practically improve patients’ outcomes or medical cost efficacy in real ED situations.

## Ethical approval

This is only a commentary, not research involving patients. No ethical approval is required.

## Consent

This is only a commentary, not research involving patients. No patient consent is required.

## Sources of funding

This is a commentary. We have no funding for our commentary.

## Author contribution

S.-U.F.C.: conceptualization and writing the original draft; S.-C.Y.: validation; C.-C.C.: conceptualization, supervision, submission, and correspondence.

## Conflicts of interest disclosure

There are no conflicts of interest in our commentary.

## Research registration unique identifying number (UIN)

This is a commentary to a study published in ‘International Journal of Surgery’. No UIN is required.

## Guarantor

Professor Chong-Chi Chiu.

## Data availability statement

This is only a commentary to a study published in ‘International Journal of Surgery’. There is no research data in our commentary.

## Provenance and peer review

This paper was not invited.
